# A Guide to De-escalation of Combination Therapy in Inflammatory Bowel Disease: A Retrospective Cohort Study

**DOI:** 10.1093/crocol/otaf026

**Published:** 2025-04-18

**Authors:** Adam A Saleh, Rajdeepsingh Waghela, Shayan Amini, Joshua Moskow, Malcom Irani, Christopher Fan, Kerri Glassner, Bincy P Abraham

**Affiliations:** Department of Internal Medicine, University of Texas Southwestern Medical Center, Dallas, TX, USA; Houston Methodist Hospital, Underwood Center for Digestive Diseases, Fondren Inflammatory Bowel Disease Center, Houston, TX, USA; Houston Methodist Hospital, Underwood Center for Digestive Diseases, Fondren Inflammatory Bowel Disease Center, Houston, TX, USA; Texas A&M College of Engineering Medicine, Houston, TX, USA; Houston Methodist Hospital, Underwood Center for Digestive Diseases, Fondren Inflammatory Bowel Disease Center, Houston, TX, USA; Houston Methodist Hospital, Underwood Center for Digestive Diseases, Fondren Inflammatory Bowel Disease Center, Houston, TX, USA; Houston Methodist Hospital, Underwood Center for Digestive Diseases, Fondren Inflammatory Bowel Disease Center, Houston, TX, USA; Houston Methodist Hospital, Underwood Center for Digestive Diseases, Fondren Inflammatory Bowel Disease Center, Houston, TX, USA

**Keywords:** combination therapy, inflammatory bowel disease, JAK inhibitors, biologic, de-escalation

## Abstract

**Background:**

Advanced combination therapy with biologics and small molecules has seen more widespread implementation for inflammatory bowel disease (IBD). However, there is a paucity of data available to guide the successful de-escalation of combination therapy following the achievement of disease remission. Therefore, we pursued this retrospective study to evaluate our center’s approach to de-escalation of these patients.

**Methods:**

IBD patients undergoing de-escalation of combination biologic therapy from May 2017 to March 2023 with a follow-up visit were included. The need for re-escalation, steroid therapy, and hospitalization at follow-up was compared between the de-escalation method, adherence, patient demographics, disease characteristics, and measures of disease activity.

**Results:**

Fifty IBD patients underwent de-escalation, with a median age of 35.7 years. All 50 patients had a follow-up visit within a median of 168 (111) days. Patients were divided into two groups with 12 (24%) patients requiring re-escalation of therapy and 38 (76%) able to maintain or further de-escalate. Of those that required re-escalation, 3 (25%) required the use of systemic steroids and none required hospitalization for IBD. Non-adherence to the de-escalation plan significantly correlated with the need for re-escalation (*P* < .001).

**Conclusions:**

Patient adherence and the number of prior failed biologic therapies were identified as potential risk factors for re-escalation. The type of agent being de-escalated (biologic or Janus kinase inhibitors [JAKi] did not correlate with the need for re-escalation).

## Graphical Abstract

**Graphical Abstract:**
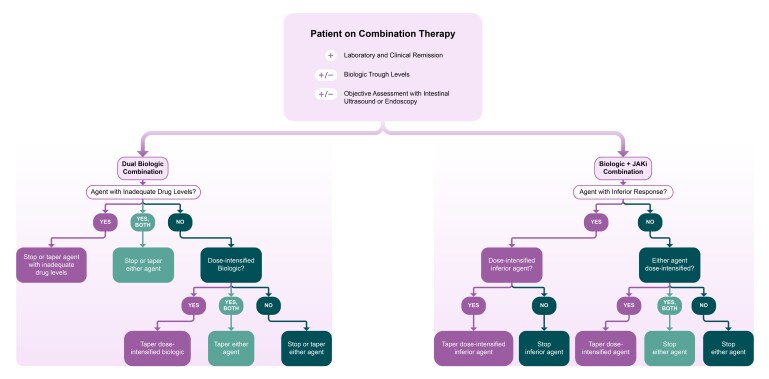

## Introduction

The treatment of moderate to severe inflammatory bowel disease (IBD) focuses on achieving mucosal healing by targeting specific immune pathways while attempting to limit long-term steroid exposure.^1^ However, given that current biologic therapy only achieves 40%-50% clinical response in 1 year, many patients with refractory diseases are left with limited options.^2-6^ In 2010, the SONIC trial was the first randomized controlled trial to assess the effectiveness of combination therapy with biologic and immunomodulatory agents for the treatment of IBD.^7^ However, the first study to evaluate the safety of dual biologic agents natalizumab and infliximab was published in 2007 and yielded positive results with patients on combination therapy achieving higher rates of clinical remission than those on biologic monotherapy.^8^ More recently, the EXPLORER trial demonstrated greater rates of clinical response with a combination of vedolizumab, adalimumab and methotrexate in Crohn’s disease (CD), and the VEGA trial showed greater clinical response in ulcerative colitis with a combination of guselkumab plus golimumab.^9,10^

With the influx of newer biologic and small molecule therapies, there has been an increasing trend towards combining medications for the management of refractory cases.^11,12^ This modality appears to be effective for inducing remission of active disease and in patients with significant extra-intestinal manifestations or concomitant rheumatologic disease.^12,13^

While there is limited data on the use of multiple biologics or JAKi in combination to treat patients with IBD, there is much less information available to guide the successful de-escalation of combination therapy once adequate response is achieved. The SPARE trial investigated the feasibility of withdrawing infliximab or concomitant immunosuppressant therapy in patients with CD on combination therapy, but immunosuppressants included were thiopurines or methotrexate.^14^

The aim of this study was to evaluate factors that lead to the successful de-escalation of dual biologic therapy or the combination of a biologic with a JAKi. We secondarily aimed to identify if specific de-escalation methods were more successful than others.

## Methods and Materials

This study was approved by the institutional review board at our institution. This retrospective analysis evaluated patients with IBD on combination therapy with 2 biologics or a biologic and JAKi who underwent de-escalation of either agent from May 2017 to March 2023. There were no patients on sphingosine-1 phosphate modulators on combination therapy. Data were collected from both the initial visit at which the decision to de-escalate was made, and the next follow-up visit. The time the patient spent on combination therapy prior to de-escalation was recorded. Laboratory results, clinical scores, and endoscopic scores within a 3-month period prior to de-escalation were included. Additional parameters were collected on the patient’s body mass index (BMI), disease characteristics, and biologics failed prior to their combination therapy. Laboratory results included albumin, C-reactive protein (CRP), erythrocyte sedimentation rate (ESR), fecal calprotectin, and fecal lactoferrin if available. Clinical scores included the Harvey-Bradshaw Index (HBI) for CD patients and the partial Mayo score and ulcerative colitis activity index (UCAI) for UC patients. Biologic drug levels were included only if they were obtained at true trough level at some point during the patient’s combination therapy regimen. Adequate drug levels were defined as follows: vedolizumab levels >20 μg/mL, ustekinumab levels >4 μg/mL, and infliximab levels >5 μg/mL (except in perianal disease in which an infliximab level of 10 μg/mL was used).^15-17^ Patients were de-escalated using one of the following methods: biologic agent de-escalation or small-molecule agent de-escalation. These categories were further subdivided into: tapering of the biologic agent, tapering of JAKi, stopping a biologic agent, and stopping a JAKi.

Laboratory results, clinical scores, and endoscopic scores obtained within 1-month of the follow-up date were included. For patients with results from both the initial and follow-up visit, the difference was calculated. As this is a retrospective study, there was no specific de-escalation protocol that was followed. However, generally, the decision to de-escalate was made in patients that had objective measures of improvement such as fecal calprotectin or with endoscopic findings or imaging findings. Tapering was done by increasing or decreasing the frequency at which biologics were given or by decreasing the dose of a JAKi.

The patient’s adherence to de-escalation plan was recorded from the follow-up visit as well. The patient was considered non-adherent if they were unable to follow de-escalation instructions for any reason. For example, if a patient were to stop taking a JAKi instead of tapering it, or if they tapered more quickly than planned, they were considered non-adherent. The need for re-escalation, re-escalation with steroid therapy, or if patients required hospitalization or surgery for their IBD was recorded.

### Statistical Analysis

Categorical variables were reported as frequencies and continuous variables as medians with interquartile range (IQR) in parentheses. The primary endpoint was the percentage of patients requiring re-escalation at follow-up visits and its association with various laboratory results, clinical scores, and endoscopies when available.

Disease phenotype, disease duration, age at diagnosis, tobacco use, time spent on combination therapy, and previously failed biologics were compared with the need for re-escalation at follow-up. Patients were grouped by the original combination therapy they were on (dual biologic therapy vs. biologic + JAKi therapy), and rates of re-escalation were compared between the different types of de-escalation. Additionally, laboratory results, clinical scores, and endoscopic scores were compared between types of de-escalation. If patients had laboratory results, clinical scores, or endoscopic evaluations at both the initial and follow-up visits, their change in values between visits was compared as well. Comparisons were made with Mann–Whitney *U* test for continuous and ordinal variables while the χ2 or the Fisher exact test was used for categorical variables. A sub-analysis was also performed on only patients whowere adherent to the de-escalation plan. Results that were significant in the sub-analysis are reported here. Results were considered statistically significant if *P* < .05 after a Bonferroni correction was applied for family-wise comparisons. Statistical analysis was conducted using R version 4.3.1 and libraries “ggplot2” and “summarytools.”

## Ethical Considerations

This research complies with the guidelines for human studies and was conducted ethically in accordance with the World Medical Association Declaration of Helsinki.

### Study Approval Statement

This study protocol was reviewed and approved by an institutional review board (IRB).

### Consent to Participate Statement

Written, informed consent was not required for this retrospective, de-identified review, confirmed by our IRB.

## Results

### Population Overview

Fifty patients met the inclusion criteria for the study. Patient and disease characteristics at the time of de-escalation are outlined in Table 1. The population had a median age of 35.7 (20.1) with 24 (48%) males and 26 (52%) females. Patients had a median BMI of 23.1 (7.2). For the disease subtype, 22 (44%) patients had CD, 25 (50%) had UC, and 3 (6%) had indeterminate colitis. Three (6%) patients were current smokers, 41 (82%) had never smoked and 6 (12%) were former smokers. Median disease duration in our population was 11 (11.8) years with a median age at diagnosis of 23.3 (14.9). For patients with CD, 8 (37%) had colonic disease, 9 (41%) had ileocolonic and 5 (22%) had upper GI and ileocolonic disease. In terms of disease behavior, 8(37%) had non-stricturing, non-penetrating disease, 6 (27%) had only stricturing disease, 2 (9%) had only penetrating disease, and 6(27%) had both stricturing and penetrating disease. 9(41%) patients had perianal disease. For patients with UC, 1 (4%) had ulcerative proctitis, 8 (32%) had left-sided UC, and 16 (64%) had pancolitis. Initial laboratory, clinical scores, endoscopic scores, medical therapy, and type of de-escalation can be found in Table 1. An exact breakdown of the agents used in combination therapy can be found in Table 2 with 15 (30%) of patients on dual biologic therapy, 32 (64%) on biologic and SM combination therapy, and 3(6%) of patients on triple combination therapy with 2 biologics and one SM agent. The median time spent on combination therapy prior to de-escalation was 13.2 (18.6) months.

**Table 1.** Descriptive overview of patient data at the time the decision to de-escalate was made. If the entire patient population was not included in the characteristic, the sample size is listed.

Characteristic	Value (total *n* = 50)
Patient age (median, IQR)	35.7 (20.1)
Male (*N*, %)	24 (48%)
Female (*N*, %)	26 (52%)
BMI (median, IQR)	23.1 (7.2)
*Smoking status*	
Current (*N*,%)	3 (6%)
Former (*N*,%)	6 (12%)
Never (*N*,%)	41 (82%)
*Disease characteristics*	
Crohn’s disease (*N*, %)	22 (44%)
Ulcerative Colitis (*N*, %)	25 (50%)
Indeterminate Colitis (*N*, %)	3 (6%)
Disease duration (median, IQR)	11 (11.8)
Age at diagnosis (median, IQR)	23.3 (14.9)
*Disease location and behavior*	
*Crohn’s disease (N = 22)*	
L1, ileal (*N*, %)	0 (0%)
L2, colonic (*N*, %)	8 (37%)
L3, ileocolonic (*N*, %)	9 (41%)
L4, isolated upper (*N*, %)	0 (0%)
L34, upper and ileocolonic (*N*, %)	5 (22%)
B1, non-stricturing, non-penetrating (*N*, %)	8 (37%)
B2, stricturing (*N*, %)	6 (27%)
B3, penetrating (*N*, %)	2 (9%)
B23, stricturing and penetrating (*N*, %)	6 (27%)
p, perianal disease (*N*, %)	9 (41%)
*Ulcerative Colitis (N = 25)*	
E1, proctitis (*N*, %)	1 (4%)
E2, left-sided (*N*, %)	8 (32%)
E3, pancolitis (*N*, %)	16 (64%)
*Previous IBD therapy*	
Biologic Naïve (*N*, %)	14 (28%)
Number of previously failed biologics (median, IQR)	1 (2)
*Laboratory results*	
Albumin (g/dL) (median, IQR)	4.1 (0.5) (*N* = 39)
CRP (mg/L) (median, IQR)	2.1 (3.6) (*N* = 36)
ESR (mm/hr) (median, IQR)	6 (17) (*N* = 37)
Fecal Lactoferrin (mcg/mL) (median, IQR)	24.6 (192) (*N* = 10)
*Clinical scores*	
HBI (median, IQR)	1.5 (6.8) (*N* = 22)
Partial Mayo (median, IQR)	0.5 (3) (*N* = 26)
UCAI (median, IQR)	1 (3) (*N* = 17)
*Endoscopic evaluation*	
Mayo score (median, IQR)	1 (1) (*N* = 12)
SES-CD (median, IQR)	1.5 (4) (*N* = 10)
*Initial medical therapy*	
Biologic + biologic (*N*, %)	15 (30%)
Biologic + SM (*N*, %)	32 (64%)
Biologic + biologic + SM (*N*, %)	3 (6%)
Concomitant prednisone use (*N*, %)	4 (8%)
Adequate biologic drug levels (*N*, %)	18 (86%)
Time on combination therapy, months (median, IQR)	13.2 (18.6)
*Type of de-escalation*	
Taper biologic (*N*, %)	17 (34%)
Stop biologic (*N*, %)	13 (26%)
Taper small molecule (*N*, %)	16 (32%)
Stop small molecule (*N*, %)	4 (8%)

N, sample size; IQR, interquartile range; BMI, body mass index; HBI, Harvey-Bradshaw Index; SM, small molecule.

**Table 2.** List of different combination therapies patients were on prior to de-escalation.

Combination	Number of patients (%)
*Biologic + biologic*	15 (30%)
Ustekinumab + Vedolizumab	14 (28%)
Adalimumab + Vedolizumab	1 (2%)
*Biologic + small-molecule*	32 (64%)
Vedolizumab + Tofacitinib	8 (16%)
Ustekinumab + Tofacitinib	8 (16%)
Infliximab + Tofacitinib	8 (16%)
Adalimumab + Tofacitinib	1 (2%)
Golimumab + Tofacitinib	1 (2%)
Infliximab + Upadacitinib	2 (4%)
Ustekinumab + Upadacitinib	2 (4%)
Risankizumab + Upadacitinib	2 (4%)
*Triple combination therapy*	3 (6%)
Ustekinumab + Vedolizumab + Tofacitinib	2 (4%)
Adalimumab + Vedolizumab + Tofacitinib	1 (2%)

All 50 patients had a follow-up visit within a median of 168 (111) days. Clinical data from the follow-up visit can be found in Table 3. Patients were divided into 2 groups with 12 (24%) patients requiring re-escalation of IBD therapy and 38 (76%) able to maintain their therapy or further de-escalate. Of those that required re-escalation, 3 (25%) required the use of systemic steroids and none required hospitalization for IBD. Prior use of corticosteroids was not associated with the need for re-escalation at follow-up with 1 out of 5 patients requiring re-escalation at follow-up (*P* = .83).

**Table 3.** Descriptive overview of patient data at the time of follow-up. If the entire patient population was not included in the characteristic, the sample size is listed. If patients had laboratory results, clinical scores, or endoscopic evaluations at both the initial and follow-up visits, their change in values between visits was compared using a Mann–Whitney *U* test. *P* values < .05 after Bonferroni correction were considered significant.

Characteristic	Value	Change from initial visit
Number of patients with follow-ups (N, %)	50 (100%)	—
Time between visits, days (median, IQR)	168 (111)	—
Patient plan compliance (*N*, %)	40 (80%)	—
BMI (median, IQR)	23.4 (5.3) (*N* = 33)	0 (.2) (*N* = 33)
*Laboratory results*		
Albumin (g/dL) (median, IQR)	4.3 (0.4) (*N* = 33)	0.1 (0.1) (*N* = 28)
CRP (mg/L) (median, IQR)	1.9 (3.8) (*N* = 32)	−3 (12) (*N* = 27)
ESR (mm/hr) (median, IQR)	6 (12) (*N* = 33)	0 (2.5) (*N* = 27)
Fecal Lactoferrin (mcg/mL) (median, IQR)	0 (11.5) (*N* = 6)	0 (*N* = 2)
*Clinical scores*		
HBI (median, IQR)	2 (4) (*N* = 19)	0 (3) (*N* = 18)
Partial Mayo (median, IQR)	0 (1.5) (*N* = 19)	0 (3) (*N* = 11)
UCAI (median, IQR)	0 (0.5) (*N* = 11)	−1 (3) (*N* = 11)
*Endoscopic evaluation*		
Mayo Score (median, IQR)	0 (1) (*N* = 5)	−1.5 (*N* = 2)
SES-CD (median, IQR)	3 (7) (*N* = 4)	1.5 (*N* = 2)
*Outcomes*		
Need for Re-escalation of Therapy (N, %)	12 (24%)	—
Required Steroids for Re-escalation (N, %)	3 (25%)	—

IQR, interquartile range; ns, non significant.; BMI, body mass index; HBI, Harvey-Bradshaw Index; SM, small molecule.

**Figure 1.**
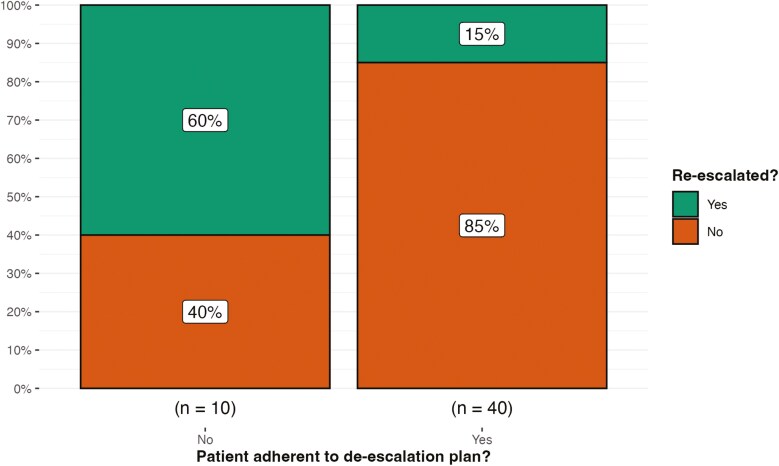
Relationship between non-adherence and the necessity for re-escalation at follow-up. Chi-squared analysis revealed a significant difference between the adherent and non-adherent populations and the need for re-escalation at follow-up (*P* < .001).

Ten (20%) of patients were not fully adherent to the initial plan for de-escalation. This was due to reasons such as sudden loss of insurance coverage and patient choice. All 3 patients requiring re-escalation with systemic steroids at follow-up were non-adherent to the de-escalation treatment plan. A full list of reasons for non-adherence can be found in Supplementary Appendix A. Chi-squared analysis revealed a significant difference between the adherent and non-adherent populations and the need for re-escalation (*P* < .001) with 60% of those that were non-adherent requiring re-escalation versus 15% in the adherent group (Figure 1).

Additionally, 17 patients had trough biologic drug levels obtained at some point during their combination regimen prior to de-escalation. Including patients on dual-biologic therapy, there were a total of 21 drug levels obtained with 18 (86%) being adequate. The 3 patients with inadequate drug levels were de-escalated by stopping their biologic medication and none of these patients required re-escalation at follow-up. Of the remaining 14 patients with adequate drug levels, 1 stopped their biologic, 5 tapered their biologic, 7 tapered their JAKi, and 1 stopped JAKi. One patient in the taper JAKi group required re-escalation at follow-up. The subtype of de-escalation within the group with drug levels was not significantly related to the need for re-escalation at follow-up (*P* = .24).

### Disease Characteristics and the Need for Re-escalation

Patients who did not require re-escalation had a median age at diagnosis of 23.9 (12) while those who did have a median age at diagnosis of 18.6 (14); although, this was not significant (*P* = .35). Median disease duration for patients who did not require re-escalation was 10.5 versus 11 for those who did (*P* = .89). Of the 3 patients who were current smokers, 2 (67%) required re-escalation while 0 out of the 6 former smokers required re-escalation and 10 (24%) of the 41 never smokers required re-escalation (*P* = .09). Sub analysis with only adherent patients did not yield significant results. Of the 22 patients with CD, 12 (45%) required re-escalation at follow-up while only 1 (4%) out of 25 patients with UC required re-escalation (*P* < .001). This remained true after subanalysis with only adherent patients (*P* < .001).

For disease phenotype, 2 (25%) of patients with colonic CD required re-escalation while 4 (44%) of patients with ileocolonic disease did and 4 (80%) patients with both upper and ileocolonic disease required re-escalation (*P* < .001). This remained true after subanalysis with only adherent patients (*P* < .001). For the 9 (41%) of CD patients with perianal disease, 3 (33%) required re-escalation versus 7 (54%) of the CD patients without perianal disease (*P* = .08). For patients with non-stricturing and non-penetrating CD, 4 (50%) required re-escalation versus 3 (50%) of patients with stricturing CD only, 1 (50%) patient with penetrating only and 2 (33%) of patients with both stricturing and penetrating CD (*P* = .45). Subanalysis with only adherent patients did not yield significant results.

### Previous Biologic Therapy and the Need for Re-escalation

**Figure 2.**
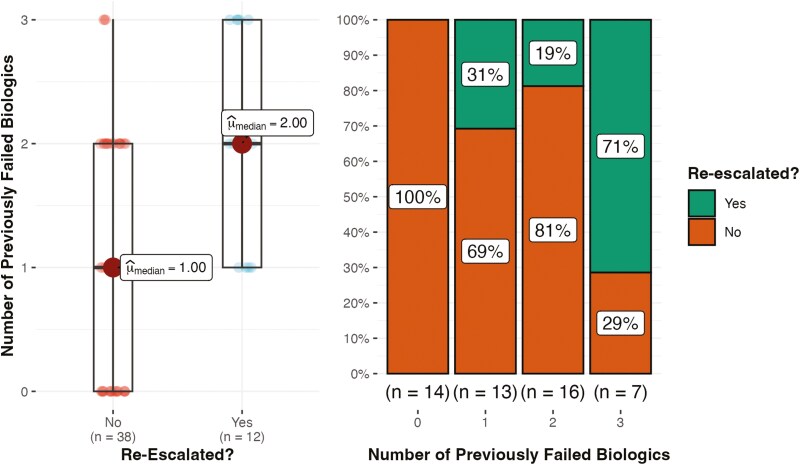
The significance of previously failed biologics on the need for re-escalation at follow-up. The median number of previously failed biologics in patients that required re-escalation was 2 (1) versus 1 (1) in patients that did not (*P* < .001). The percent of patients requiring re-escalation grouped by the exact number of previously failed biologics is also shown.

The median time spent on combination therapy prior to de-escalation for patients that required re-escalation was 13.6 (5) months versus 12.7 (10) months for patients who did not (*P* = .82). Patients who were biologically naïve had a median time spent on combination therapy of 8.2 (15.1) months versus 13.5 (19.6) months for patients with previous biologic failures (*P* = .14). Out of the 14 patients who were biologically naïve prior to their current IBD regimen, zero required re-escalation at follow-up versus 33% of patients with previously failed biologics (*P* = .01). Additionally, none of the biologically naïve patients required the use of corticosteroids during their treatment course prior to de-escalation. The median number of previously failed biologics in patients who required re-escalation was 2 (1) versus 1 (1) in patients who did not (*P* < .001; Figure 2). This remained true after subanalysis with only adherent patients (*P* < .001). When further subdivided into the exact number of previously failed biologics, 71% of patients with 3 previous biologic failures required re-escalation at follow-up versus 19% of 2 and 31% for 1 previous failure (Figure 2). Subanalysis with only adherent patients did not yield significant results.

### Changes in Clinical Data Association With Re-escalation of IBD Therapy

The median changes in values for patients with repeat laboratory tests, clinical scores, or endoscopy scores between visits can be found in Table 3. Patients who required re-escalation of medical therapy did not have a statistically significant difference in changes in their BMI, albumin, CRP, ESR, and lactoferrin levels. Nor did they have a statistically significant difference in the changes in their clinical scores (HBI, partial Mayo, and UCAI). There were not enough repeat endoscopies for statistical comparisons. Subanalysis with only adherent patients did not yield significant results.

### Initial Clinical Data and the Need for Re-escalation at Follow-up

#### Clinical scores

After Bonferroni correction was applied, the median HBI at the initial visit in which the decision to de-escalate was made was 2 (*N* = 17) for those who did not require re-escalation and 0 (*N* = 5) for those who did (*P* = .87). For UC patients, median partial Mayo score at the initial visit was 1 (*N* = 19) for patients who did not require re-escalation and 0 (*N* = 7) for patients who did (*P* = .85). The median UCAI score was 1 (*N* = 14) for patients who did not require re-escalation and 0 (*N* = 3) for those that did (*P* = .42).

#### Laboratory markers

**Figure 3.**
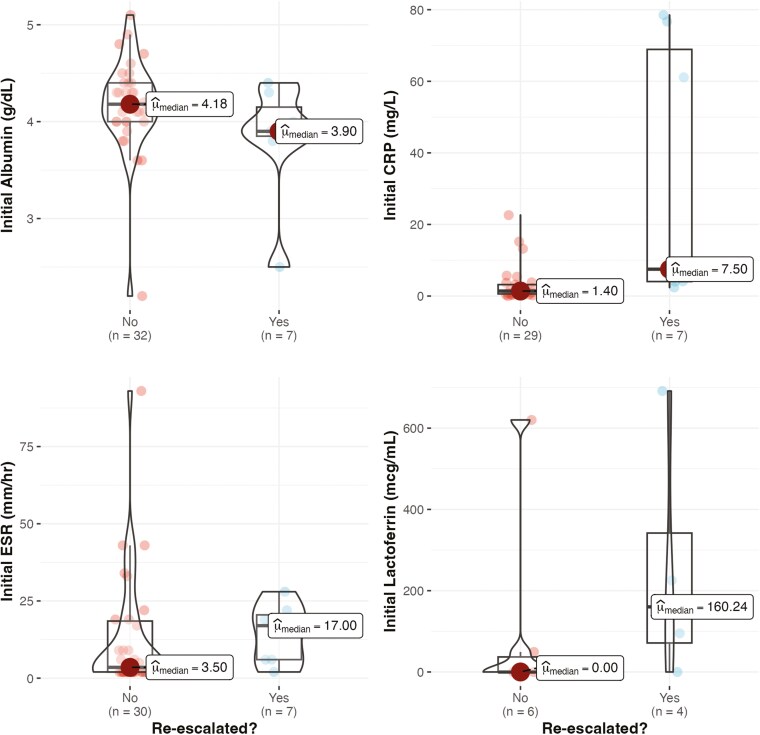
Boxplot with superimposed violin plot for laboratory marker values at the initial visit in which the decision to de-escalate was made. Patients are grouped based on the need for re-escalation at follow-up and were compared via Mann–Whitney U comparison. After Bonferroni correction, the initial CRP was significantly higher (7.5 vs. 1.4 mg/L) in patients that required re-escalation at follow-up (*P* < .001).

As can be seen in Figure 3, the median albumin at the initial visit was 3.9 g/dL (*N* = 7) in patients who required re-escalation versus 4.18 g/dL in those who did not (*P* = .11). The initial CRP was significantly higher (7.5 vs. 1.4 mg/L) in patients who required re-escalation at follow-up (*P* < .001 with Bonferroni correction). Median ESR was 17 mm/hr (*N* = 7) in patients who required de-escalation versus 3.5 mm/hr (*N* = 30) in those who did not (*P* = .22). Finally, median lactoferrin was 160.24 mcg/mL (*N* = 4) for patients who required re-escalation versus 0 mcg/mL (*N* = 6) for patients who did not (*P* = .21).

The overall difference in clinical scores and inflammatory markers between the initial visit and follow-up was not significant. These results can be found in Supplementary Appendix B, C, and D.

### Type of De-escalation Association With Re-escalation of IBD Therapy

#### Dual biologic combination therapy

**Figure 4.**
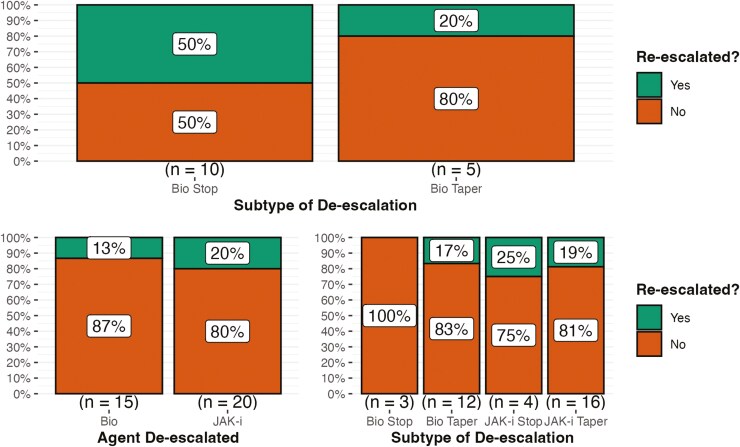
Agent de-escalated association with the need for re-escalation at follow-up grouped by original therapy with (A) being patients originally on dual-biologic therapy and (B) being patients on combination therapy with a biologic agent and JAK-inhibitor. Subtype of De-escalation was not significantly associated with the need for re-escalation at follow-up in both groups (A) and (B) (*P* = .26, .84, respectively); although, tapering either biologic or JAK-inhibitors trended towards lower rates of re-escalation than stopping either agent. In group (B), there was no significant difference between de-escalating a biologic agent versus a JAK-inhibitor (*P* = .6).

Out of the 15 patients on combination therapy with dual biologic agents, biologic agents were tapered in 5 (33%) of our patients while 10 (67%) stopped their biologic therapy. As shown in Figure 4A, 50% of patients who stopped their biologic required re-escalation at follow-up while only 20% of patients who tapered required re-escalation; however, chi-squared analysis revealed no statistical difference between the need for re-escalation in the 2 subgroups (*P* = .26). Sub analysis with only adherent patients did not yield significant results.

There was no significant difference between patients who stopped versus tapered their biologic and resulting changes in albumin, CRP, ESR, and IBD clinical scores between the initial visit and follow-up. Subanalysis with only adherent patients did not yield significant results.

#### Biologic and JAKi combination therapy

Out of the 35 patients on combination therapy with at least one biologic and a JAKi, biologic agents were de-escalated in 15 (43%) of our patient population with 12 (34%) tapering their biologic therapy and 3 (9%) stopping their biologic therapy. JAKi therapy was de-escalated in 20 (57%) of patients with 16 (46%) tapering their JAKi therapy and 4 (11%) stopping JAKi therapy. As shown in Figure 4B, Chi-squared analysis revealed no statistical difference between the need for re-escalation in the biologic de-escalation group versus the JAKi de-escalation group (13% vs. 20%, respectively, *P* = .60). There was no significant difference (*P* = .84) between the rates of re-escalation between those who stopped biologic therapy (0%), tapered biologic therapy (17%), stopped JAKis (25%), and tapered JAKis (19%). Subanalysis with only adherent patients did not yield significant results.

There was no significant difference between the agent de-escalated and resulting changes in albumin, CRP, ESR, and IBD clinical scores between the initial visit and follow-up. This remained true for the specific subtype of de-escalation as well (tapering versus stopping the agent). Subanalysis with only adherent patients did not yield significant results.

## Discussion

In this retrospective cohort study, de-escalation of combination therapy was successful in 76% of patients at follow-up after a median duration of 168 (111) days. Of the patients who had to be re-escalated at follow-up, only those who were non-adherent with the de-escalation plan required systemic steroids and none were hospitalized for IBD. As such, when controlling for patient adherence, de-escalation of combination therapy in our population did not result in increased use of systemic steroids. Patients who were non-adherent with the treatment plan faced significantly higher rates of re-escalation than their adherent counterparts. This suggests that there should be extra attention placed on the patient’s understanding of the treatment plan prior to commencing with de-escalation.

Additionally, our results showed that 100% of patients who were naïve to biologic therapy prior to those included in their combination treatment plan did not require re-escalation of IBD therapy at their follow-up visit (*P* < .001). Importantly, these patients were also able to avoid the use of systemic corticosteroids to achieve clinical remission and were then successfully de-escalated. Of these patients, 13 out of 14 were on combination therapy with a biologic and JAK inhibitor. This might suggest that the use of JAK inhibitors in combination with a biologic can be used in the short term to avoid steroid use; however, further investigation in larger sample sizes is required.

Patients who required re-escalation at follow-up had a median number of 2 previously failed biologics while patients with 71% of patients with 3 previously failed biologics required re-escalation. In this case, the number of previously failed biologics is likely suggestive of how refractory a patient’s disease activity is and the necessity for combination therapy in the first place. This is further supported by the fact that disease subtype was significantly correlated with the need for re-escalation with 45% of CD patients requiring re-escalation versus only 4% of UC (*P* < .001). We also found that patients were significantly more likely to require re-escalation at follow-up if they had concomitant upper GI and ileocolonic CD. Our results suggest that previous biologic failures and disease subtypes could be used to risk-stratify patients prior to de-escalation of their combination therapy.

We additionally found that the need for re-escalation did not trend with specific changes in laboratory results, or clinical scores. However, initial laboratory markers appear to be an important consideration in the decision to de-escalate. Patients who required re-escalation had lower albumin levels and higher ESR, lactoferrin, and CRP levels with CRP reaching significance. It is possible that ESR and lactoferrin not reaching significance is a type II error given the relatively small sample size of patients who required re-escalation; however, this is not completely unexpected given that biomarkers are not always sensitive to disease severity and extent.^18,19^

Similarly, both changes in clinical score and initial clinical scores did not correlate with the need for re-escalation. Thus we do not recommend initiation of de-escalation based on clinical scores alone. However, this is not necessarily unexpected given that HBI scores do not always correlate accurately with disease activity.^20,21^ Given that there was only one UC patient who required re-escalation at follow-up, analysis of the change in UC clinical scores and its association with re-escalation was not possible. However, the median change in clinical scores for the UC patients at follow-up was 0 for the partial Mayo score and −1 for the UCAI score, suggesting that these patients were tolerating de-escalation well.

All but 3 patients had adequate drug levels at de-escalation. These 3 patients were de-escalated by stopping the biologic with inadequate drug levels and did not require re-escalation at follow-up. This suggests that drug levels can be used to identify which agent to safely de-escalate first although our sample size is limited. The type of de-escalation did not significantly correlate with the necessity for re-escalation with 27% of biologic de-escalation and 20% of JAKi de-escalation requiring re-escalation at follow-up. This was true for the specific subtype as well (tapering an agent vs. stopping an agent); although, for both biologic de-escalation and JAKi de-escalation, the rates of re-escalation were greater in patients who stopped either therapy versus tapering (38% vs. 18% for biologic de-escalation and 25% and 19% for JAKi de-escalation).

In general, the decision to de-escalate was more likely to be successful when there were some objective markers of improvement in inflammation. Practically, this can be seen with an improved fecal calprotectin, endoscopic remission/mild disease or signs, decreased wall thickness or Doppler activity on IUS, or improvement in inflammation on imaging with computed tomography enterography or magnetic resonance enterography. Regarding tapering versus stopping combination therapy, we would recommend first tapering if the patient is on a dose-intensified regimen of either small molecule or biologic therapy.

Our retrospective cohort study was limited by our low number of patients with repeat fecal inflammatory markers and endoscopic scores within the time frame required for inclusion in this study, making it more difficult to quantify disease activity. Additionally, this study did not investigate the effects of de-escalating specific medications, and we instead used larger classifications of biologics and JAKis due to our sample size. As such, future studies should seek to investigate larger cohorts in a prospective manner to reduce the number of missing values for analysis. It would also be useful for additional studies to follow patients who required re-escalation and observe if they were able to achieve clinical remission without significant morbidity.

From a clinical perspective, this study provides real-world evidence that patients can successfully de-escalate therapy if they require multiple advanced therapies for the treatment of their IBD. This is beneficial information for our patients to know when we are initiating combination therapy, and also to be provided to insurance payors that the escalation to more than one therapy does not have to be permanent and successful de-escalation can occur if the patient is able to remain adherent to the appropriate de-escalation plan.

## Conclusion

De-escalation of combination therapy in patients with IBD was successful in 76% of patients at follow-up with patient adherence, the number of previously failed biologic therapies, and CD being possible risk stratifiers for the need for re-escalation. The need for re-escalation at follow-up was not associated with the type of agent being de-escalated (biologic vs. JAKis).

## Supplementary Material

otaf026_suppl_Supplementary_Materials

otaf026_suppl_Supplementary_Materials_12

## Data Availability

The data underlying this article will be shared on reasonable request to the corresponding author.

## References

[1] Peyrin-Biroulet L , Sandborn W, Sands BE, et al Selecting therapeutic targets in inflammatory bowel disease (STRIDE): determining therapeutic goals for treat-to-target. Am J Gastroenterol. 2015;110(9):1324-1338. doi: https://doi.org/10.1038/ajg.2015.233 26303131

[2] Feagan BG , Rutgeerts P, Sands BE, et al; GEMINI 1 Study Group. Vedolizumab as induction and maintenance therapy for ulcerative colitis. N Engl J Med. 2013;369(8):699-710. doi: https://doi.org/10.1056/NEJMoa1215734 23964932

[3] Sandborn WJ , Feagan BG, Rutgeerts P, et al; GEMINI 2 Study Group. Vedolizumab as induction and maintenance therapy for crohn’s disease. N Engl J Med. 2013;369(8):711-721. doi: https://doi.org/10.1056/NEJMoa1215739 23964933

[4] Sandborn WJ , Gasink C, Gao LL, et al; CERTIFI Study Group. Ustekinumab induction and maintenance therapy in refractory crohn’s disease. N Engl J Med. 2012;367(16):1519-1528. doi: https://doi.org/10.1056/NEJMoa1203572 23075178

[5] Sands BE , Peyrin-Biroulet L, Loftus EV, et al; VARSITY Study Group. Vedolizumab versus adalimumab for moderate-to-severe ulcerative colitis. N Engl J Med. 2019;381(13):1215-1226. doi: https://doi.org/10.1056/NEJMoa1905725 31553834

[6] Sands BE , Sandborn WJ, Panaccione R, et al; UNIFI Study Group. Ustekinumab as induction and maintenance therapy for ulcerative colitis. N Engl J Med. 2019;381(13):1201-1214. doi: https://doi.org/10.1056/NEJMoa1900750 31553833

[7] Colombel JF , Sands BE, Rutgeerts P, et al The safety of vedolizumab for ulcerative colitis and Crohn’s disease. Gut. 2017;66(5):839-851. doi: https://doi.org/10.1136/gutjnl-2015-311079 26893500 PMC5531223

[8] Sands BE , Kozarek R, Spainhour J, et al Safety and tolerability of concurrent natalizumab treatment for patients with Crohnʼs disease not in remission while receiving infliximab. Inflamm Bowel Dis. 2007;13(1):2-11. doi: https://doi.org/10.1002/ibd.20014 17206633

[9] Colombel JF , Ungaro RC, Sands BE, et al Vedolizumab, adalimumab, and methotrexate combination therapyin crohn’s disease (EXPLORER). Clin Gastroenterol Hepatol. 2023;22(7):1487-1496.e12. doi: https://doi.org/10.1016/j.cgh.2023.09.010 37743037

[10] Feagan BG , Sands BE, Sandborn WJ, et al; VEGA Study Group. Guselkumab plus golimumab combination therapy versus guselkumab or golimumab monotherapy in patients with ulcerative colitis (VEGA): a randomised, double-blind, controlled, phase 2, proof-of-concept trial. Lancet Gastroenterol Hepatol. 2023;8(4):307-320. doi: https://doi.org/10.1016/S2468-1253(22)00427-7 36738762

[11] Abreu MT. Combining biologic agents in inflammatory bowel disease. *Gastroenterol Hepatol.* 2019;15(10):549-551. PMC6883734 31802979

[12] Glassner K , Oglat A, Duran A, et al The use of combination biological or small molecule therapy in inflammatory bowel disease: a retrospective cohort study. J Dig Dis. 2020;21(5):264-271. doi: https://doi.org/10.1111/1751-2980.12867 32324969

[13] Ahmed W , Galati J, Kumar A, et al Dual biologic or small molecule therapy for treatment of inflammatory bowel disease: a systematic review and meta-analysis. Clin Gastroenterol Hepatol. 2022;20(3):e361-e379. doi: https://doi.org/10.1016/j.cgh.2021.03.034 33798711

[14] Louis E , Resche-Rigon M, Laharie D, et al; GETAID and the SPARE-Biocycle research group. Withdrawal of infliximab or concomitant immunosuppressant therapy in patients with Crohn’s disease on combination therapy (SPARE): a multicentre, open-label, randomised controlled trial. Lancet Gastroenterol Hepatol. 2023;8(3):215-227. doi: https://doi.org/10.1016/S2468-1253(22)00385-5 36640794 PMC9908559

[15] Ansari M , Glassner K, Irani M, et al Therapeutic drug monitoring in inflammatory bowel disease patients on vedolizumab. J Dig Dis. 2024;25(2):91-99. doi: https://doi.org/10.1111/1751-2980.13261 38599667

[16] Saleh A , Stading R, Miroballi N, Glassner K, Abraham BP. Therapeutic drug monitoring in patients with inflammatory bowel disease on ustekinumab. J Dig Dis. 2024;25(4):214-221. doi: https://doi.org/10.1111/1751-2980.13264 38587053

[17] Abraham BP , Ziring D, Dervieux T, Han PA, Shim A, Battat R. Real-world impact of infliximab precision-guided dosing on management of patients with IBD. Am J Manag Care. 2023;29(suppl 12):S227-S235. doi: https://doi.org/10.37765/ajmc.2023.89447 37844322

[18] Schoepfer AM , Vavricka S, Zahnd-Straumann N, Straumann A, Beglinger C. Monitoring inflammatory bowel disease activity: clinical activity is judged to be more relevant than endoscopic severity or biomarkers. J Crohns Colitis. 2012;6(4):412-418. doi: https://doi.org/10.1016/j.crohns.2011.09.008 22398068

[19] Halpin SJ , Ford AC. Prevalence of symptoms meeting criteria for irritable bowel syndrome in inflammatory bowel disease: systematic review and meta-analysis. Am J Gastroenterol. 2012;107(10):1474-1482. doi: https://doi.org/10.1038/ajg.2012.260 22929759

[20] Gomes P , du Boulay C, Smith CL, Holdstock G. Relationship between disease activity indices and colonoscopic findings in patients with colonic inflammatory bowel disease. Gut. 1986;27(1):92-95. doi: https://doi.org/10.1136/gut.27.1.92 3949241 PMC1433178

[21] Sandborn WJ , Feagan BG, Hanauer SB, et al A review of activity indices and efficacy endpoints for clinical trials of medical therapy in adults with Crohn’s disease. Gastroenterology. 2002;122(2):512-530. doi: https://doi.org/10.1053/gast.2002.31072 11832465

