# Supplementary material for: A Guide to De-escalation of Combination Therapy in Inflammatory Bowel Disease: A Retrospective Cohort Study
Source: Crohns Colitis 360. 2025 Apr 18;7(2):otaf026. doi: 10.1093/crocol/otaf026 (PMC12048838; doi:10.1093/crocol/otaf026)

**Appendix A.** Table with full list of reasons for why patients were non-adherent with their de-escalation treatment plan (N=10). Patients are listed in chronological order in which they began de-escalation.

| **Patient** | **Reason for non-adherence** |
| --- | --- |
| **Patient 1** | Stopped taking tofacitinib instead of tapering. |
| **Patient 2** | Lapse in insurance coverage for vedolizumab for unknown reason. |
| **Patient 3** | Lapse in insurance coverage for vedolizumab for unknown reason. |
| **Patient 4** | Stopped taking tofacitinib instead of tapering. |
| **Patient 5** | Patient was incorrectly taking 40 mg of tofacitinib instead of labeled instructions to take 10 mg. |
| **Patient 6** | Lapse in insurance coverage for ustekinumab for unknown reason. |
| **Patient 7** | Patient decision to stop taking vedolizumab. |
| **Patient 8** | Stopped taking tofacitinib instead of tapering. |
| **Patient 9** | Lapse in insurance coverage for ustekinumab for unknown reason. |
| **Patient 10** | Stopped taking tofacitinib instead of tapering. |

SM = Small Molecule

**Appendix B:** Boxplot with superimposed violin plot of laboratory markers taken at the time the decision to de-escalate was made and those collected at follow-up. Initial laboratory values collected were compared with follow-up laboratory values using a Mann-Whitney U test comparison. No laboratory values were significantly different after a Bonferroni correction was applied.

Please note a high quality figure has been separately uploaded. It’s included below as well for your convenience.


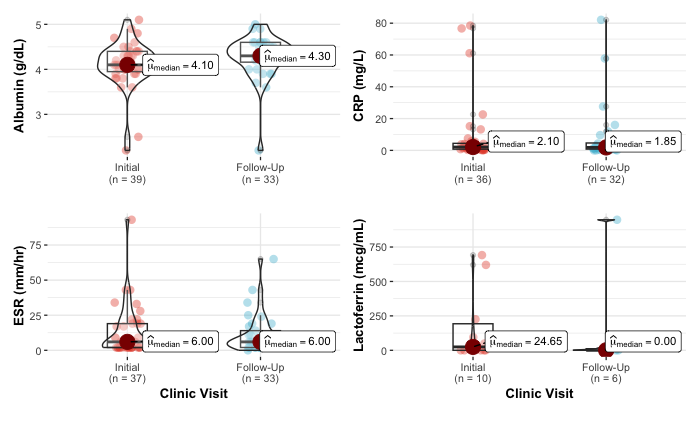


**Appendix C:** Boxplot with superimposed violin plot of inflammatory bowel disease clinical scores taken at the time the decision to de-escalate was made and those collected at follow-up. Initial clinical scores were compared with follow-up clinical scores using a Mann-Whitney U test comparison. No clinical scores were significantly different after a Bonferroni correction was applied.

Please note a high quality figure has been separately uploaded. It’s included below as well for your convenience.


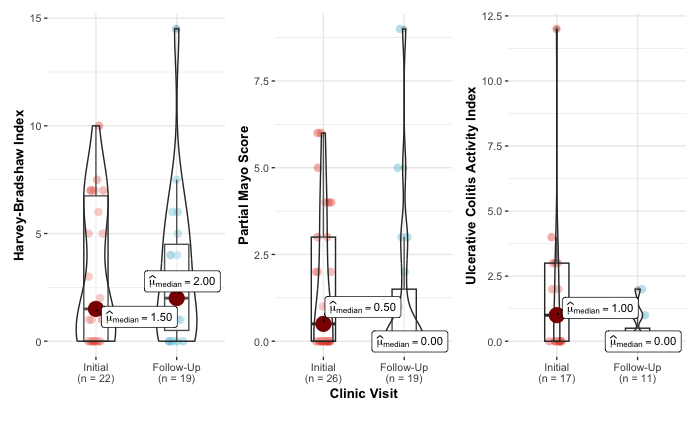


**Appendix D:** Boxplot with superimposed violin plot of inflammatory bowel disease endoscopic scores taken within 3 months of the decision to de-escalate and those within 3 months of the follow-up date. Initial endoscopic scores were compared with follow-up endoscopic scores Mann-Whitney U test comparison. No endoscopic scores were significantly different after a Bonferroni correction was applied.

Please note a high quality figure has been separately uploaded. It’s included below as well for your convenience.


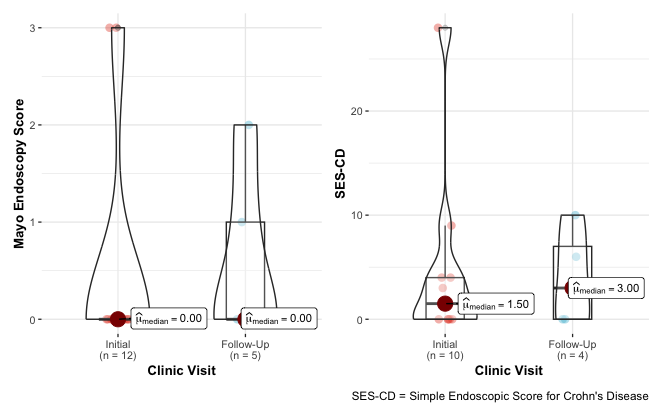

Supplement: otaf026_suppl_Supplementary_Materials [file otaf026_suppl_supplementary_materials.docx]
