# Supplementary figures and images for: A Guide to De-escalation of Combination Therapy in Inflammatory Bowel Disease: A Retrospective Cohort Study
Source: Crohns Colitis 360. 2025 Apr 18;7(2):otaf026. doi: 10.1093/crocol/otaf026 (PMC12048838; doi:10.1093/crocol/otaf026)

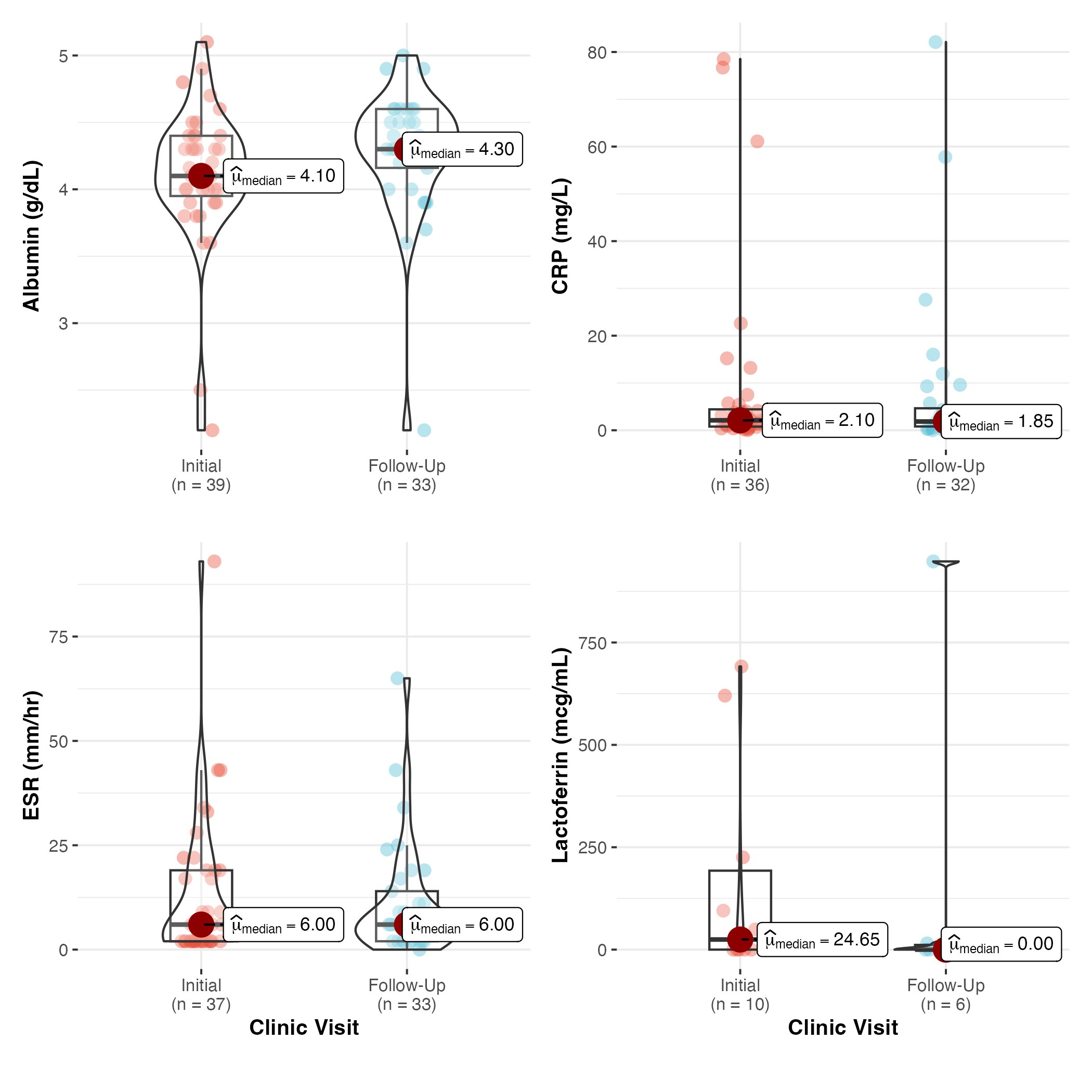

Supplement: otaf026_suppl_Supplementary_Materials_12 [file otaf026_suppl_supplementary_materials_12.jpeg]
